# Regeneration of the Sciatic Nerve

**Published:** 1850-06

**Authors:** 


### SELECTIONS

FROM

### AMERICAN AND FOREIGN JOURNALS.

---

*Regeneration of the Sciatic Nerve.*—M. Brown Séquard recently exhibited to the Biological Society of Paris a Guinea-pig, in which the sciatic nerve had been completely divided twelve months previously. A month after the operation, indications of a return of sensibility had begun to present themselves; within another month, the sensibility had decidedly augmented, but was still much inferior to that of the sound limb; the muscles had then begun again to act. Six months after the section, the sensibility appeared to have returned completely, and the motor power to have been almost entirely recovered; and at the end of about eleven months, no difference could be discovered in the motor power of the two limbs. The animal having been subsequently killed, a careful examination was made of the part of the nerve where the section had been made; and the restoration of the original state was so complete, that no indication of the division could be discovered, either with the naked eye, or with the microscope. The usual swelling at the point of reunion had been distinguishable up to about the sixth month; but it had then disappeared.—*B. and For. Med. Chir. Rev.*, Jan., 1850, from *Gazette Médicale de Paris*, No. 45.

